# Puppies as the primary causal animal for human rabies cases: three-year prospective study of human rabies in the Philippines

**DOI:** 10.3389/fmicb.2024.1425766

**Published:** 2024-07-08

**Authors:** Nobuo Saito, Karren L. Inton, Jaira D. Mauhay, Rontgene M. Solante, Ferdinand D. Guzman, Kentaro Yamada, Yasuhiko Kamiya, Mariko Saito-Obata, Beatriz P. Quiambao, Takaaki Yahiro, Kazunori Kimitsuki, Akira Nishizono

**Affiliations:** ^1^Research Center for Global and Local Infectious Diseases, Oita University, Yufu, Japan; ^2^Department of Microbiology, Faculty of Medicine, Oita University, Yufu, Japan; ^3^Institute of Tropical Medicine, Nagasaki University, Nagasaki, Japan; ^4^School of Tropical Medicine and Global Health, Nagasaki University, Nagasaki, Japan; ^5^San Lazaro Hospital, Manila, Philippines; ^6^Laboratory of Veterinary Public Health, Department of Veterinary Medical Science, Faculty of Agriculture, University of Miyazaki, Miyazaki, Japan; ^7^Tohoku University Graduate School of Medicine, Sendai, Japan; ^8^Research Institute for Tropical Medicine, Muntinlupa, Philippines

**Keywords:** rabies, puppy, human rabies, the Philippines, post-exposure prophylaxis (PEP), PEP, vaccines

## Abstract

**Introduction:**

While rabies remains a global concern, detailed studies on human rabies, particularly regarding causal animals and the reasons for not receiving postexposure prophylaxis (PEP), are lacking.

**Methods:**

We conducted a 3-year prospective study (October 2019–September 2022) at the Philippines’ largest rabies referral center. We interviewed patients with suspected rabies and their families. We used LN34 qRT-PCR and rapid fluorescent focus inhibition test on saliva samples. We also compared our findings with two retrospective studies at the same hospital.

**Results:**

We enrolled 151 patients, including 131 with potential rabies exposure. Similar to retrospective studies, the participants were predominantly males (75.5%), adults (76.8%), low-income individuals (91.4%), and rural dwellers (62.3%). The causal animals were mainly dogs (97.0%), with similar incubation periods, clinical symptoms, and a high proportion not receiving vaccines or immunoglobulins (93.2%). Most causal animals were owned by either the patients’ households or their neighbors (60.2%), with a significant proportion being puppies (58.8%). Most patients had knowledge of rabies; however, reasons for not seeking PEP included misconceptions about minor bites not causing rabies (51.3%), beliefs in traditional healers (33.9%), and economic constraints (22.6%). Despite completing the WHO regimen, two PEP failures were observed. LN34 qRT-PCR detected 98 positive cases (sensitivity, 64.9%; 95% CI 56.7–72.5). These strains belong to the Southeast Asia 4 subclade.

**Discussion:**

In conclusion, this study highlights the role of puppies as primary causal animals and the presence of misconceptions that preclude patients from acquiring PEP.

## 1 Introduction

Rabies is a highly infectious and almost always fatal disease (Hemachudha et al., 2013). In developing countries, it remains a major public health concern, with domestic dogs as the primary source of transmission to humans and animals (WHO, 2018; WHO, FAO, and OIE, 2018). Human rabies can be prevented with post-exposure prophylaxis (PEP), which includes wound cleaning, rabies immunoglobulin administration, and vaccination. The “Zero by 30” initiative aims to eradicate dog-mediated rabies deaths by 2030, but global rabies incidence has not significantly decreased despite the initiative (WHO, FAO, and OIE, 2018; Nadal et al., 2023).

The Philippines introduced an intradermal regimen and expanded its treatment network for PEP, but rabies cases remain unchanged (Supplementary Figure 1; Quiambao et al., 2005; National Rabies Prevention and Control Program in the Philippines, 2019). Limited availability of rabies vaccines for animals and inadequate preventive strategies for free-roaming dogs contribute to this issue. The rapidly increasing populations of humans and pet animals in recent years have led to a rise in human-animal interactions and animal bites, making the provision of adequate rabies vaccines and rabies immunoglobulin (RIG) for PEP increasingly challenging (Dizon et al., 2022). Over one million individuals in the Philippines receive PEP annually, resulting in a considerable economic burden for the country (Amparo et al., 2018b).

A significant issue is the limited understanding of human rabies risk factors due to a lack of prospective studies. Previous studies mainly relied on retrospective designs or national surveillance data, which had limitations in describing the causal animals and factors associated with not receiving PEP (Dimaano et al., 2011; Guo et al., 2018; Vargas et al., 2019; Ghosh et al., 2020; Rana et al., 2020; Duarte et al., 2021; Guzman et al., 2022). Two retrospective studies in the Philippines analyzed 1,839 patients from 1978 to 2006 and 437 patients from 2006 to 2015. These studies showed that only 1.7 and 9.6% of patients, respectively, received a rabies vaccine (Dimaano et al., 2011; Guzman et al., 2022). However, comprehensive data on reasons for not seeking PEP were unavailable due to the retrospective nature of the study. A community study in the Philippines demonstrated that although there was a high level of awareness and knowledge about rabies and the locations of health facilities where PEP can be received (Amparo et al., 2018a,b). Despite this, only 44.9% of individuals sought PEP after animal exposure. The primary reasons for not seeking PEP were “Did not know I needed to go” (37.4%), “No money” (22.7%), and “Not a severe wound” (19.9%). While this study identified reasons why the general population did not receive PEP, the reasons for not receiving PEP among human rabies patients have not been examined. Identifying detailed risk factors for human rabies can help develop targeted educational strategies and allocate resources efficiently. To address these issues, a detailed analysis of human rabies patients using prospective data collection is necessary. This study aims to provide comprehensive information on human rabies cases in the Philippines, including the causal animals and reasons for not receiving PEP.

## 2 Materials and methods

### 2.1 Ethics statement

Ethical approval was obtained from the Institutional review board of San Lazaro Hospital (SLH) (SLH-RERU-2019-016-E) and Oita University (No. 1457). For patients older than 18 years who could express their intentions, we obtained written informed consent from the patient.

In cases where the patient was unconscious, written consent was provided by a family member or relative. We obtained informed consent from both the family member and the patient with altered mental status or those aged < 18 who could express their intentions. For young children under 5, we obtained written consent from their families.

### 2.2 Study sites

SLH is located in Manila City, National Capital Region (NCR; also called Metro Manila) and is the largest national referral center for rabies and other notifiable infectious diseases in the Philippines. It admits 60 to 80 rabies cases annually. From 2007 to 2022, SLH admitted approximately 20% of human rabies cases in the country (Supplementary Figure 2). The hospital serves patients from a wide catchment area, encompassing roughly a 100 km radius, including NCR, Regions III, and IV-A. Most human rabies cases are clinically diagnosed, and the hospital does not provide intensive care such as mechanical ventilation with rabies. A final diagnosis of rabies was made at the hospital discharge if the patient exhibited distinct rabies symptoms, including hydrophobia and aerophobia and died within a few days of admission.

### 2.3 Study flow

We conducted a three-year prospective observational study enrolling patients with suspected rabies admitted to SLH from 1 October 2019 to 30 September 2022. Upon arrival of a suspected rabies patient to the emergency room, the research team was notified and approached both the patient and their family. We enrolled clinically diagnosed rabies by an attending physician if they showed signs of rabies dominated by forms of hyperactivity, including aerophobia and hydrophobia, or paralytic syndrome. After obtaining consent, the research team interviewed patients and their families using a structured questionnaire. Following admission, the research team monitored the patients’ condition daily and extracted details regarding treatment and outcomes from medical records.

### 2.4 Analysis flow

**FIGURE 1:**
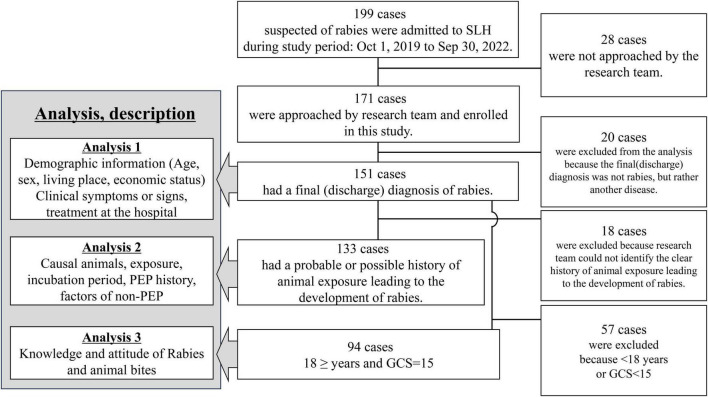
Flow chart of the inclusion for each analysis in this study. SLH, San Lazaro Hospital; GCS, Glasgow Coma Scale; PEP, post-exposure prophylaxis.

The analysis comprised three steps (Figure 1). First, we described the demographic data, pet ownership, medical history, clinical findings, and hospital treatments (Analysis 1). Second, we categorized the animal exposure history into 5 classifications: “Definite,” “Probable,” “Possible,” “Doubtful,” and “Unknown” to exclude cases with unclear or unrelated animal exposure to rabies (The definitions were provided in Supplementary Table 1). This category was created based on the WHO rabies animal case definitions for this study. We only included cases with “Definite,” “Probable,” and “Possible” exposure history in the analysis of the causal animal, animal exposure, patient post-exposure responses, and reasons for not receiving rabies vaccine and immunoglobulin (Analysis 2). Lastly, we interviewed and analyzed the knowledge and attitudes toward rabies and animal bites in patients > 17 years old with intact consciousness (Glasgow Coma Scale [GCS] = 15) (Analysis 3) (Supplementary Table 2). We compared the data from the present study with data from two previous retrospective studies at SLH (1987–2006 [*n* = 1839] and 2006–2011 [*n* = 463]).

### 2.5 Samples and laboratory methods

During enrolment, blood samples and an initial saliva sample were collected, followed by two additional sequential saliva samples obtained at least one hour apart. If the patient was unable to produce saliva, saliva swabs were collected (Supplementary material pages 6–7). We performed RNA extraction, LN34 RT-qPCR, and partial nucleoprotein (*N*) gene sequencing to generate a phylogenetic tree with genetic clades assigned by RABV-GLUE^1^ (Supplementary material pages 8–12, Supplementary Table 3) (Cai et al., 2011; Wadhwa et al., 2017; Mauhay et al., 2023). We also performed a rapid fluorescent focus inhibition test (RFFIT) to detect the presence of virus-neutralizing antibodies in the patient’s serum. Less than 0.05 IU/mL was considered undetectable.

### 2.6 Data collection and management

Study data were collected and managed using REDCap electronic data capture tools hosted at Nagasaki University (REDCap Consortium, Nashville, TN, USA). Statistical analyses were performed using Stata software (Stata Statistical Software Release 17; Stata Corp., College Station, TX, USA). We performed a one-sample chi-squared test to compare the proportions of living area, assuming a null probability of 0.5. We converted the home locations and exposure sites of rabies patients into Global Positioning System (GPS) coordinates and used Geographic Information System (GIS) software (ArcGIS Pro version 3.2; ESRI, Redlands, CA, USA) for mapping purposes. Case maps were generated using data from the exposure site, if available; otherwise, residence location was used if the exposure history or site was unknown. We conducted a comparative analysis of case distributions in the current study by comparing them to case maps and heatmaps from a retrospective study from 2006 to 2015, which visually represented the density of human rabies cases using kernel density analysis. Definitions of rural and urban areas were adopted in accordance with those provided by the Philippine Statistics Authority.

## 3 Results

During the study, 199 suspected rabies patients were admitted to SLH. Overall, 28 patients with a final rabies diagnosis were not included due to staff unavailability (Figure 1 and Supplementary Figure 3). Of the included 171 patients, 151 were diagnosed with rabies at discharge, and 20 survived with other diseases (Analysis 1). Among the 151 patients, the likelihood of animal exposure leading to rabies was classified as follows: 0 cases as “Definite,” 55 as “Probable,” 78 as “Possible,” 5 as “Doubtful,” and 13 as “Unknown.” Analysis 2 included 133 cases with “Probable” and “Possible” animal exposures. Analysis 3 involved 94 adults with clear consciousness for rabies knowledge analysis.

### 3.1 Demographics of human rabies cases

**FIGURE 2:**
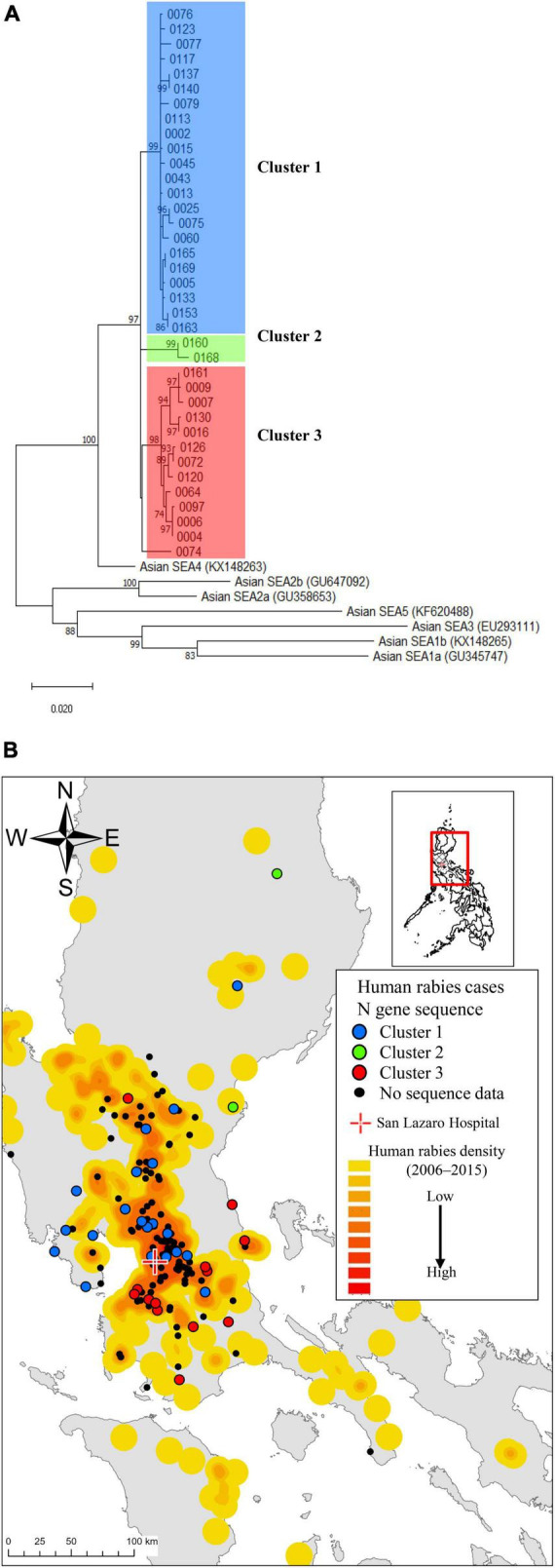
Spatial distributions of human rabies cases and the rabies virus strains by *N* sequence clusters. **(A)** A phylogenetic tree constructed from 37 rabies-positive samples revealed three genetic clusters based on the *N* gene sequence. **(B)** The case distribution of human rabies cases and the *N* gene sequence clusters admitted to San Lazaro Hospital (red cross) from October 2019 to September 2022 is shown in **(B)**. The case map is overlaid on a heatmap representing the case density of previous rabies cases from 2006 to 2015, with a gradient from low (yellow) to high (red) density. Geographical plot of the *N* gene sequence clusters with color-coded points (Blue, Green, Red and Black dot) aligned with the clusters identified in **(A)**. The baseline map was taken from the United Nations Office for the Coordination of Human Affairs (https://data.humdata.org/dataset/philippines-administrative-levels-0-to-3).

The annual average (mean) number of human rabies cases during the study period was 60.0, which was nearly the same as that observed between 2006 and 2015 (Supplementary Table 4). While male patients predominated, with adult males comprising over half (*n* = 86, 57.0%), 35 (23.2%) were younger than 20 years (Table 1). More patients lived in rural areas (94, 62.3%) than in urban areas (57, 37.7%) (*P* < 0.01). The majority of patients were from Region III (69, 45.7%), followed by Region IV-A (53, 35.1%) and the NCR (28, 18.5%) (Supplementary Figure 2). Most of the exposure sites were near residential locations, with only seven cases at distances greater than 10 km. The case maps indicate that the locations of the patients in the current study were similar to those identified in the retrospective study between 2006 and 2015 (Figure 2B). Many were low-income households with low educational attainment. Most patients (102, 67.7%) had dogs or cats. Among the 102 pet owners, 80 (78.4%) allowed their pets to roam freely, and only 15 (14.7%) had vaccinated their pets against rabies. No patients received immunosuppressive therapy.

**TABLE 1:** Characteristics of 151 patients with a final diagnosis of rabies at discharge.

		*N* (%)
Age	Under 5	3 (2.0)
	5–19 years	32 (21.2)
	20–29 years	19 (12.6)
	30–39 years	26 (17.2)
	40–49 years	29 (19.2)
	Over 50	42 (27.8)
Sex	Male	114 (75.5)
	Female	37 (24.5)
Living area	Rural	94 (62.3)
	Urban	57 (37.7)
Monthly household income (PHP/month)	< 5,000	45 (29.8)
	5,000–10,000	64 (42.4)
	10,001–20,000	29 (19.2)
	> 20,000	12 (7.9)
	Unknown	1 (0.7)
Ownership of dog or cat pets	Yes	102 (67.5)
	No	49 (32.5)
Pets allowed to roam freely outside (*N* = 102)	Yes	80 (78.4)
	No	22 (21.6)
Rabies vaccination status of their dog or cat pets (*N* = 102)	None vaccinated	61 (59.8)
	All pets vaccinated	15 (14.7)
	At least one pet vaccinated, but not all	25 (24.5)
	Unknown	1 (1.0)
Animal exposure histories leading to rabies	Definite	0 (0)
	Probable	55 (36.4)
	Possible	78 (51.7)
	Doubtful	5 (3.3)
	Unknown	13 (8.7)

All patients were deceased at the time of discharge. San Lazaro Hospital is located in the national capital city. PHP, Philippine Peso (1 PHP = 0.018 USD).

### 3.2 Clinical features

Most patients exhibited hydrophobia (*n* = 151, 100%) and aerophobia (145, 96.0%). Additionally, 42 (27.8%) patients had a fever, and 21 (13.9%) were in shock at the time of admission (Supplementary Table 5). Most patients had intact consciousness, but 36 (23.3%) had a low GCS score (< 15) upon arrival at the ER. All patients were diagnosed with furious rabies. The median incubation period from exposure to symptom onset was 61 days (range 10–1,052 days), with 91 cases (68.4%) having an incubation period of 90 days or less and four cases (3.0%) exceeding 365 days (Supplementary Figure 4). The incubation period tended to be shorter in patients with bites on the face or neck (Supplementary Table 6). The median duration from symptom onset to admission was 2 days (range, 0–6 days), and from onset to death was 3 days (range, 0–8 days). Overall, 98.3% died within 48 h of admission (Supplementary Table 5).

### 3.3 Laboratory results

LN34 qRT-PCR using a maximum of three serial saliva or saliva swab samples detected 98 positive cases with 64.9% sensitivity (56.7–72.5) and 100.0% specificity (83.2–100.0), using patients with a final diagnosis of rabies as the reference group (Supplementary Tables 7, 8). When calculated per number of samples, the sensitivity for the first specimen alone was 46.4% (95% confidence interval [CI]: 38.2–54.6). However, the sensitivity increased to 59.7% (95% CI 51.2–67.8) when two specimens were combined, and further improved to 67.4% (95% CI 59.0–75.0) when three specimens were available. All 37 sequence-positive strains belonged to the Southeast Asia 4 (SEA4) subclade, and three major clusters were observed (Figure 2A). The case map showed regional variations in the three clusters (Figure 2B). Among 126 cases with collected blood samples, 12 patients without prior rabies vaccine history showed increased antibody levels, with 11 in the 0.05–0.499 IU/mL range and one over 0.5 IU/mL (Supplementary Table 9). The antibody levels of patients receiving three or more vaccine doses after rabies-related animal exposures were ≥ 0.5 IU/mL, while those receiving one or two doses had levels below the detectable threshold (< 0.05 IU/mL).

### 3.4 Characteristics of causal animals and the animal exposures

Of the 133 patients with a probable or possible rabies-related bite history, dogs (*n* = 129, 97.0%) were the identified causal animals, followed by cats (4, 3.0%) (Table 2). Most of these animals were owned by either the patients’ households (51, 38.4%) or their neighbors (29, 21.8%). Of the 68 dogs with recorded ages, the majority were puppies, with 27 (39.7%) aged < 3 months and 13 (19.1%) aged 4–11 months. Numerous cases either found the causal animals dead (44, 33.1%) or killed (48, 36.1%) within 10 days after the bite incidence, yet none were submitted for confirmatory testing (Supplementary Table 10). Most animal exposures were transdermal bites (121, 91.0%), although there were a few cases of scratches (5, 3.8%) or licking open wounds (1, 0.8%). Of the animal bites, 12.8% were on the face or neck, and 42.1% were on the hands.

**TABLE 2:** Characteristics of causal animals, exposures, and post-exposure prophylaxis among 133 patients with probable or possible animal exposure leading to rabies.

		*N* (%)
Animal type	Dog	129 (97.0)
	Cat	4 (3.0)
Age of the animal (months) (*n* = 68, 65 were unknown age)	1–3 months	27 (39.7)
	4–11 months	13 (19.1)
	12–23 months	15 (22.1)
	≥ 24 months	13 (19.1)
Owner of the animals	Owned by the patient’s household	51 (38.4)
	Owned by neighbor	29 (21.8)
	Community-owned	3 (2.3)
	Roaming, owned by somebody else	5 (3.8)
	Stray/roaming without owner	44 (33.1)
	Unknown*	1 (0.8)
Characteristics of the exposure	Licking or nibbling on uncovered skin	1 (0.8)
	Transdermal scratches	5 (3.8)
	Transdermal bites	121 (91.0)
	Other	1 (0.8)*
Type of exposure	Multiple	41 (30.8)
	Single	92 (69.2)
Body part	Upper limb	11 (8.3)
	Lower limb	50 (37.6)
	Hands	56 (42.1)
	Head or neck	17 (12.8)
**Characteristics of post-exposure prophylaxis and health-seeking behavior**		
Wound washing	None	14 (10.5)
	Water only	2 (1.5)
	Soap and water	109 (82.0)
	Alcohol only	6 (4.5)
	Soap, water, and alcohol	2 (1.5)
	Iodine	0 (0.0)
Duration of wound washing with water	< 5 min	56 (49.6)
	5–14 min	40 (35.4)
	15 min over	8 (7.1)
	Unknown	9 (8.0)
Attending traditional healers	Yes	44 (33.1)
	No	89 (66.9)
Attending medical facilities after the animal exposure	No	115 (86.5)
	Animal bite treatment center (ABTC)	7 (5.3)
	Hospital	8 (6.0)
	Public clinic	2 (1.5)
	Private clinic	1 (0.8)
Rabies immunoglobulin (RIG) + vaccination	None	124 (93.2)
	RIG + 3 vaccinations	3 (2.3)
	RIG + 2 vaccinations	1 (0.8)
	RIG + 1 vaccination	0 (0)
	3 Vaccinations only	3 (2.3)
	2 Vaccinations only	1 (0.8)
	1 Vaccination only	1 (0.8)

*The patient provided care for the dog, which was suspected of having rabies and was later found dead. RIG, rabies immunoglobulin.

### 3.5 Characteristics of post-exposure prophylaxis and health-seeking behavior

**FIGURE 3:**
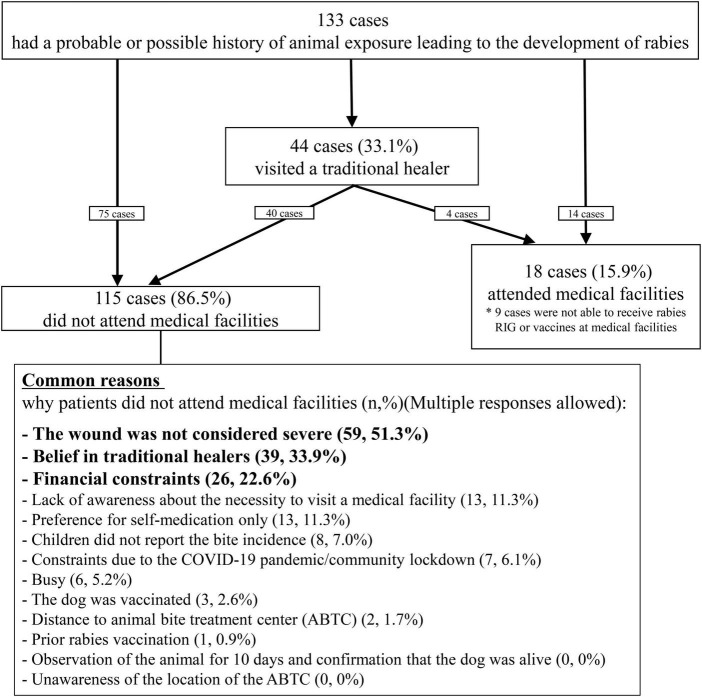
Responses of 133 patients following the animal exposures and reasons for not seeking rabies prophylaxis at medical facilities.

Although 89.5% of the patients cleaned their bite sites, the majority (85.0%) cleaned them for less than 15 min (Table 2). Some patients incorrectly treated their wounds at home, such as by applying garlic (23, 17.3%) or sucking on the wound (50, 37.6%). A total of 44 (33.1%) patients visited a traditional healer. Of the 44 patients, 4 subsequently visited the medical facility. Only 18 patients (13.5%) sought treatment at medical facilities, but among them, nine did not receive vaccines or RIG due to the unavailability of vaccines or RIG in the health facilities or patients‘ affordability (Figure 3 and Supplementary Table 11). Consequently, among 131 patients who had animal bite exposures, 124 (93.2%) patients did not receive either the vaccine or the RIG, and of these, 115 (87.8%) did not visit a medical facility for PEP. Only nine patients (6.8%) received at least one dose of rabies vaccine after the bite exposure. Of these nine, two patients (1.5%) completed PEP with three doses of rabies vaccine and RIG in accordance with the WHO recommended regimen (Table 2).

### 3.6 Reasons for not seeking medical cares

The most common reason for not seeking medical care was the misconception that animal bite injuries, being minor and not severe enough to cause rabies, rendered visits to medical facilities unnecessary (59, 51.3%). Among the 59 cases, 33 were aware of the age of the causal animal, and 23 (69.9%) were puppies. Other common reasons included beliefs in traditional medicine (39, 33.9%) and financial constraints (26, 22.6%). A small proportion (13, 11.3%) were unaware of the need for medical treatment. Eight children (7.0%) failed to report the animal bite to their parents or guardians and subsequently did not receive PEP. Seven patients (6.1%) were unable to attend medical facilities due to the COVID-19 pandemic and community lockdowns (Supplementary Table 12). A few patients (2, 1.7%) reported distance to the facilities as the reason for not seeking treatment, whereas none cited a lack of knowledge about the location of health facilities or ABTC where PEP was provided (0, 0%).

### 3.7 PEP failures

Out of the nine patients who received at least one dose of a rabies vaccine, two patients were suspected of having PEP failure despite completing the WHO-recommended regimen (Table 3). Two patients, a 61-year-old male (Study ID 1) and a 6-year-old male (Study ID 49), had short incubation periods of 12 and 17 days, respectively, after facial dog bite injuries. Upon admission, antibody levels were elevated, and saliva RT-qPCR tests were negative. One patient, a three-year-old boy (Study ID 20), had a head bite but did not receive RIG. His rabies antibody level was high (20.63 IU/mL). Two other patients, Study IDs 31 and 65, received only two and one dose of the vaccine, respectively, and showed undetectable antibody levels.

**TABLE 3:** Characteristics and treatment profiles of rabies patients receiving vaccine and immunoglobulin after the animal bite exposure.

Study ID	Age	Sex	Causal animal	Bite type*	Body part	Treatment RIG and rabies vaccination*	Incubation period (days)**	Days after exposure vaccine given	Days after exposure RIG was given	Vaccine brand	Type of RIG	RIG injection route	Dose of RIG	Saliva LN34 RT-PCR	RFFIT (IU/mL)
1	61	Male	Dog	Multiple	Face, lower limb	RIG (Day 0) + 3 Vac (Day 0, 4, 7)	17	1	1	Speeda	ERIG	Infiltration	–	Negative	5.64
49	6	Male	Dog	Single	Face	RIG (Day 1) + 3 Vac (Day 0, 3, 7)	12	0	1	Rabipur	ERIG	Infiltration	–	Negative	2.8
167	23	Male	Dog	Single	Lower limb	RIG (Day 8) + 3 Vac (Day 0, 3, 8)	27	1	9	Vaxirab	ERIG	IM	12.6	Negative	0.84
166	40	Male	Dog	Single	Hand	RIG (Day 0) + 2 Vac (Day 0, 3)	41	0	0	Vaxirab	ERIG	IM	8.6	Positive	No test
20	3	Male	Dog	Single	Face	3 Vac only (Day 0, 4, 7)	14	0		Verorab				Negative	20.63
46	62	Male	Dog	Single	Hand	3 Vac only (Day 0, 4, 7)	21	0		–				Negative	0.96
119	6	Male	Dog	Single	Face	3 Vac only (Day 0, 3, 8)	17	0		Vaxirab				Negative	24.64
65	53	Male	Dog	Multiple	Lower limb	2 Vac only (Day 0, 3)	347	0		–				Positive	< 0.05
31	36	Female	Cat	Single	Lower limb	1 Vac Only (Day 0)	109	2		–				Negative	<0.05

*The first day of vaccination was defined as day 0. All bites listed in the table were classified as WHO category III. **The incubation period is defined as the number of days from exposure to the onset of initial symptoms of rabies. –, Unknown. RIG, rabies immunoglobulin. ERIG, equine RIG. Vac, vaccine/vaccines. IM: intramuscular injection. RFFIT, rapid fluorescence focus inhibition test.

### 3.8 Patient knowledge about rabies

Among the patients who responded to the questions, a significant majority (94 patients, 94.4%) had knowledge of rabies and its mode of transmission (97.9%). Additionally, a substantial proportion of the patients knew the importance of washing the wound (86, 89.6%), the need to seek medical attention at health facilities such as ABTC (74, 77.1%), and were knowledgeable about the location of the nearest ABTC (97, 97.9%) (Table 4).

**TABLE 4:** Rabies patients’ knowledge and practices following animal bite exposure (*n* = 96).

		*N* (%)
Rabies knowledge	Known collectively	94 (97.9)
	Unknown or incorrect answer	2 (2.1)
Transmission	Through bites or scratches	94 (97.9)
	Unknown or incorrect answer	2 (2.1)
Casual animal	Dog	95 (99.0)
	Cat	95 (99.0)
Actions following dog bites (Multiple responses allowed)	Do nothing	1 (1.0)
	Wash wound	86 (89.6)
	Consult traditional healer	16 (16.7)
	Apply traditional remedies at home (garlic/stone/papaya)	11 (11.5)
	Confine animal for observation	1 (1.0)
	Visit the animal bite treatment center	74 (77.1)
	Request rabies test on the animal	2 (2.1)
	Euthanize the animal	8 (8.3)
Knowledge of animal bite treatment center (ABTC)	Correctly identified nearest location	94 (97.9)
	Aware of ABTC but the location is not known	1 (1.0)
	Unaware of ABTC	1 (1.0)

## 4 Discussion

This 3-year, large-scale, prospective, observational study on human rabies has provided critical insights into rabies prevention in humans. It is the first report to use systematic prospective data collection to highlight puppies as a marked cause of human rabies. The study elucidated the key factors behind the non-receipt of PEP, including misconceptions about minor animal injuries as the primary factor, followed by belief in traditional healers and financial constraints. Additionally, the study identified two potential cases of PEP failure among 151 human rabies cases.

### 4.1 Characteristics of human rabies

Similar to the previous retrospective studies, we found a higher rabies incidence among adult males and patients from low-income backgrounds (Dimaano et al., 2011; Guzman et al., 2022). The annual numbers did not show a notable decline, and cases were continuously observed in similar geographical areas. The clinical symptoms and incubation periods were similar to those observed in previous studies, and all patients were diagnosed with furious rabies (Dimaano et al., 2011; Guzman et al., 2022). The continued occurrence of rabies in these areas may indicate a lack of improvement in preventive measures, particularly vaccination and other preventative efforts targeting dogs, over the past 20 years. The persistence of similar viral strains in these areas suggests sustained local transmission. Strengthening intervention efforts by national and local governments in these regions is crucial.

### 4.2 Puppy as the primary casual animal

During our interviews, we found that a significant number of causal animals were either patients’ pets or pets of nearby residents (60.2%). Most of these animals were puppies. The prevalence of pet dogs as causal animals is similar to reports from China (Ren et al., 2015; Guo et al., 2018) but different from Bangladesh, where stray dogs are the most common causal animals (Ghosh et al., 2020; Rana et al., 2020). In the Philippines, many households keep dogs as pets and allow them to roam freely, but a significant number of dogs are not vaccinated (Mananggit et al., 2021). Previous studies on human rabies have not provided much information on the age of animals causing human rabies (Song et al., 2009; Dimaano et al., 2011; Susilawathi et al., 2012; Ren et al., 2015; Mani et al., 2016; Guo et al., 2018; Vargas et al., 2019; Ghosh et al., 2020; Rana et al., 2020; Duarte et al., 2021; Guzman et al., 2022). A study in the Philippines showed that the incidence of canine rabies was highest among puppies, with 38.0% under 3 months old and 24.0% between 3 and 11 months old (Mananggit et al., 2021). There was a tragic case of a tourist from Norway dying of rabies after being bitten by puppies in the Philippines (WHO Rabies Information System, 2019). Further studies are necessary to determine the cause of the high incidence of rabies in puppies. The demographic data of puppy is lacking in rabies-endemic countries, and epidemiological studies are needed to assess the prevalence of the disease within the puppy and adult dog population. Detailed investigations into puppy rabies cases, examining the sources of infection and the health status of their mothers and siblings, are also necessary to clarify why rabies is more prevalent among puppies. Puppies may cause mild bite incidents more frequently than adult dogs. These animal behaviors of puppy are also contributing factor. The major contributing factor is the ineffective puppy vaccination strategy in countries with endemic dog-mediated human rabies. The vaccination rate of puppies is presumed to be very low across the country. Mass rabies vaccination campaigns for domestic animals are typically conducted once a year, usually in March during Rabies Awareness Month, and not during other months. In these campaigns, puppies under 3 months old are not vaccinated, so many puppies born during the campaign remain unvaccinated until the following year’s campaign (National Rabies Prevention and Control Program in the Philippines, 2019). There is an urgent need to improve puppy vaccination efforts and revise vaccination regimens for infants under 3 months old, as some studies have shown that vaccination is effective in this age group (Morters et al., 2015; Arega et al., 2020; Kazadi Kawaya et al., 2022). Considering the need for multiple vaccine doses to achieve sufficient antibody levels in infants, oral vaccines may be a convenient option. Furthermore, it is essential to implement preventive measures that restrict contact between unvaccinated puppies and other dogs, and to increase the frequency of mass animal vaccinations of rabies to twice a year.

### 4.3 Common reasons not seeking PEP

Furthermore, this interview revealed common reasons for rabies patients not seeking PEP in health facilities. Previous studies have shown that most Filipinos are knowledgeable about rabies, and our study found the same in human rabies patients (Amparo et al., 2018a; Dizon et al., 2022). Despite adequate knowledge regarding rabies and the location of health facilities that provide PEP, many patients do not seek treatment. The primary reason is that they perceive mild bite injuries as insignificant and not requiring vaccination, often caused by puppies. This misconception requires targeted awareness campaigns. Other reasons include beliefs in traditional healers and economic constraints, consistent with community survey findings in the Philippines (Amparo et al., 2018a). Some people choose traditional medicine because it is affordable and accessible. Children accounted for a smaller proportion of human rabies cases, despite having a higher incidence of bites in the community. This may be due to children seeking medical care from their families (Amparo et al., 2018a,b). Some individuals did not receive proper treatment due to community lockdowns during the COVID-19 pandemic and vaccine shortages at healthcare facilities. These cases should be reviewed to improve the healthcare system (Supplementary Table 11).

### 4.4 PEP failure

In our previous retrospective study, two cases of PEP failure were reported, but limited information was available on RIG administration (Dimaano et al., 2011; Guzman et al., 2022). In our current analysis of 151 cases, we found two cases of PEP failure (1.3%). Further investigations, including direct inquiries with healthcare facilities, confirmed proper PEP administration in these cases at the health facilities, and antibody levels increased during admission. Although the WHO PEP regimen is considered nearly 100% effective, there have been occasional reports of PEP failure (Guzman et al., 2022; Whitehouse et al., 2023). PEP failure is more likely in children or those with severe facial bites, as observed in our two cases. In these cases, antibody titers increased upon admission, and saliva RT-PCR results were negative. Administering RIG to the face, particularly in children, can be challenging and may result in inadequate treatment. In cases of facial bites, there is a higher likelihood of the virus entering the nerves directly before antibodies are induced by vaccination (Whitehouse et al., 2023). Although the exact number of individuals who received PEP in the catchment areas (NCR, Region 3, and Region 4) were unknown in our study, approximately 100,000 people annually receive PEP for WHO category 3 animal exposures, indicating that the incidence of rabies is considered to be very rare (National Rabies Prevention and Control Program in the Philippines, 2019). A recent systematic review disclosed 122 cases of breakthrough infections (Whitehouse et al., 2023). A research conducted in Cambodia analyzed 1,739 individuals bitten by rabid dogs, identifying three potential instances PEP failure, corresponding to a rate of 0.17% (95% Confidence Interval: 0.03–0.50) (Tarantola et al., 2019).

Our study highlights the rare possibility of PEP failure with the current WHO regimen. Additional treatments, such as antiviral therapy, should be evaluated urgently in cases of severe bites to reduce the risk, as some drugs have shown antiviral effects (Yamada et al., 2016).

### 4.5 Limitation

This study has some limitations. This study was conducted at a single healthcare facility in a specific geographic area, which may limit the generalizability of the findings to a broader population with different healthcare infrastructures and cultural practices. Brain biopsy, which is a definitive diagnostic method, was not performed due to challenges in obtaining consent, potentially resulting in missed or misdiagnosed cases. However, patients diagnosed with rabies in this study exhibited typical symptoms, such as hydrophobia, and a rapidly progressing clinical course leading to a fatal outcome. Therefore, there is a high level of confidence in the presence of rabies in these cases. We have adhered to the STROBE guidelines in our study (Supplementary Table 13).

## 5 Conclusion

Our study highlights the need to raise awareness about the risk of rabies, especially from bites by puppies and minor injuries. To decrease human rabies cases, we should strengthen puppy vaccination programs and educate individuals about the misconception surrounding minor animal bites.

## Data availability statement

The datasets presented in this study can be found in online repositories. The names of the repository/repositories and accession number(s) can be found in this article/Supplementary material.

## Ethics statement

The studies involving humans were approved by the San Lazaro Hospital (SLH) (SLH-RERU-2019-016-E) and Oita University (No. 1457). The studies were conducted in accordance with the local legislation and institutional requirements. Written informed consent for participation in this study was provided by the participants’ legal guardians/next of kin.

## Author contributions

NS: Conceptualization, Data curation, Formal analysis, Investigation, Methodology, Project administration, Software, Validation, Visualization, Writing – original draft, Writing – review & editing. KI: Investigation, Methodology, Project administration, Writing – review & editing. JM: Investigation, Methodology, Project administration, Visualization, Writing – original draft, Writing – review & editing. RS: Conceptualization, Project administration, Resources, Supervision, Writing – review & editing. FG: Conceptualization, Project administration, Resources, Supervision, Writing – review & editing. KY: Data curation, Formal analysis, Validation, Writing – review & editing. YK: Data curation, Formal analysis, Methodology, Supervision, Writing – review & editing. MS-O: Data curation, Formal analysis, Supervision, Writing – review & editing. BQ: Conceptualization, Supervision, Writing – review & editing. TY: Data curation, Formal analysis, Project administration, Resources, Validation, Writing – review & editing. KK: Data curation, Formal analysis, Investigation, Project administration, Software, Validation, Writing – original draft, Writing – review & editing. AN: Conceptualization, Data curation, Formal analysis, Funding acquisition, Investigation, Supervision, Validation, Writing – review & editing.

## Footnotes

1
http://rabv-glue.cvr.gla.ac.uk
