# Supplementary material for: Puppies as the primary causal animal for human rabies cases: three-year prospective study of human rabies in the Philippines
Source: Front Microbiol. 2024 Jul 8;15:1425766. doi: 10.3389/fmicb.2024.1425766 (PMC11260713; doi:10.3389/fmicb.2024.1425766)
Supplement: Supplementary file 1 [file Data_Sheet_1.PDF]

Supplementary materials

Table of Contents

Supplementary Figure S1. Yearly number of human rabies cases in the Philippines and patients admitted at San Lazaro Hospital between 2007 and 2022. 2

Supplementary Figure S2. Location of San Lazaro Hospital and Administrative map of the National Capital Region, Region III, and Region IV-A in the Philippines. 3

Supplementary Table S1. Definitions of likelihood categories of animal exposures leading to rabies. 4

Supplementary Table S2. Questioner of Knowledge, attitudes, and practices of patient 5

Laboratory methods. Standard Operation Procedure (SOP) 6

SOP. Collection saliva or swab samples from rabies suspected patients 6

SOP. RNA extraction from saliva or swab samples 8

1.1 SOP N sequencing method 9

Supplementary Figure S3. Monthly rabies admissions and study enrollments at San Lazaro Hospital during the study period 13

Supplementary Table S4 Comparison between two previous retrospective studies (1987-2006, 2006-2011) and the prospective study (October 2019 - September 2022). 14

Supplementary Table S5 Clinical symptoms, hospital treatments, and intervals from illness onset to hospital outcomes among rabies patients with a final diagnosis of rabies (n=151) 15

Supplementary Figure S4 17

Distribution of incubation periods among rabies patients with a history of animal exposure (n=133) 17

Supplementary Table S6. Relationship between incubation period and bitten body parts 18

Supplementary Table S7. Sensitivity and specificity of LN34-qRT-PCR using single, double, or triple saliva/swab samples. 19

Supplementary Table S8. Sensitivity and specificity by sample type (Saliva or Swab) of LN34 RT-qPCR 20

Supplementary Table S9. Rapid Fluorescent Focus Inhibition Test (RFFIT) results by the frequencies of rabies vaccinations 21

Supplementary Table S10. Additional information of Table 2. Outcomes of the biting animals, rabies tests, wrong home treatments, and treatments by traditional healer 22

Supplementary Table S11 Reasons patients did not receive rabies vaccination and immunoglobulin at medical facilities. 23

Supplementary Table S12 Covid19 pandemic related factors and reasons patients were unable to visit health facilities. 25

Supplementary Table S13. STROBE Statement—checklist of items that should be included in reports of observational studies. 26

Supplementary Figure S1.
Yearly number of human rabies cases in the Philippines and patients admitted at San Lazaro Hospital between 2007 and 2022.

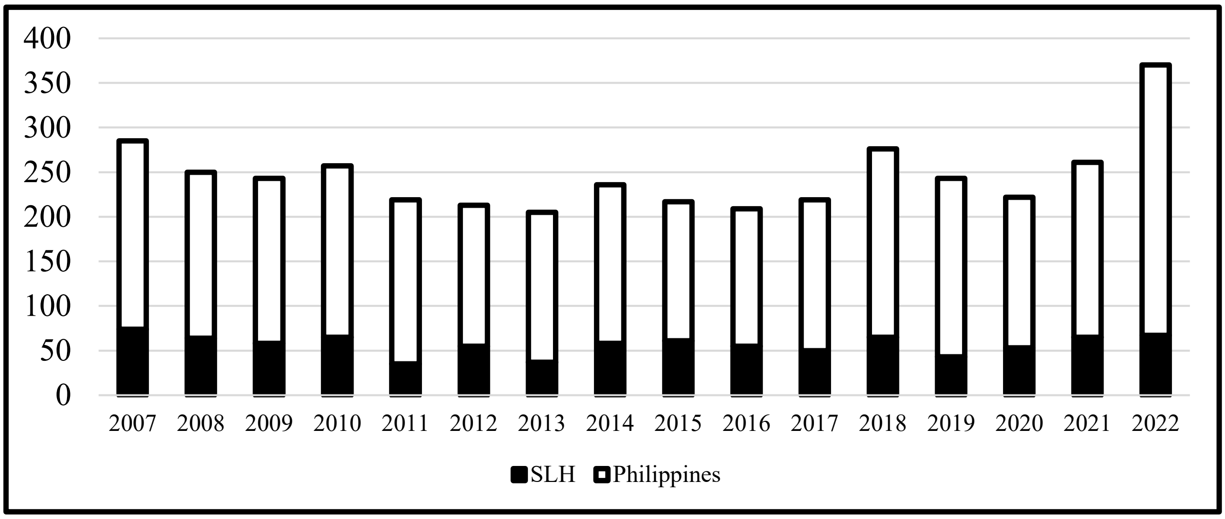

The graph displays the annual number of rabies cases in the Philippines on the vertical axis, with the number of patients admitted to San Lazaro Hospital specifically indicated in black. The number of patients admitted to San Lazaro Hospital accounts for 23.1% (905/3925) of the total national rabies cases from 2008 to 2022.

Reference:

1. Philippines Department of Health. National Rabies Prevention and Control Program-Manual of Operations (2012) | Department of Health website. https://doh.gov.ph/node/5697. Retrieved 10 June 2021.
2. Department of Health, Philippines. Rabies Surveillance report. https://doh.gov.ph/taxonomy/term/4795. Retrieved 1 November 2023.
3. National Rabies Prevention and Control Program in the Philippines. Manual of Pcocedures (2019). https://doh.gov.ph/sites/default/files/publications/Rabies%20Manual_MOP_2019%20nov28.pdf. Retrieved 10 June 2021.

Supplementary Figure S2.
Location of San Lazaro Hospital and Administrative map of the National Capital Region, Region III, and Region IV-A in the Philippines.

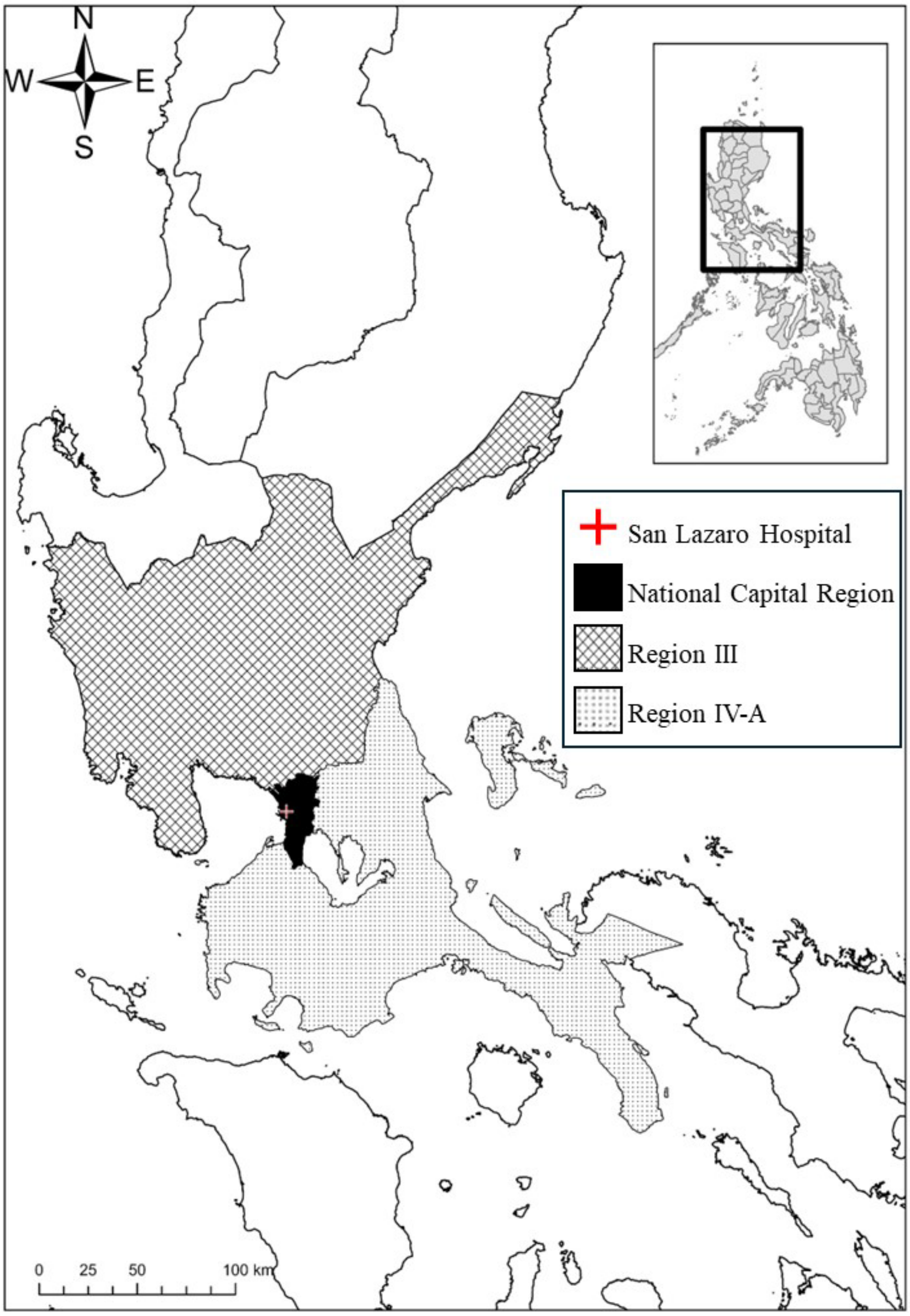

The baseline map and regional boundary data were taken from the United Nations Office for the Coordination of Human Affairs (OCHA). (https://data.humdata.org/dataset/philippines-administrative-levels-0-to-3).

Supplementary Table S1.
Definitions of likelihood categories of animal exposures leading to rabies.

Categories	Definition	WHO, Animal case definition*
"Definite"	The biting animal confirmed by laboratory tests	Confirmed
"Probable"	The biting animal died or was killed/euthanized within 14 days post-bite and showing following rabies signs.<br>- Hypersalivation<br>- Paralysis<br>- Lethargy<br>- Unprovoked abnormal aggression (biting two or more people or animals and/or inanimate objectives)<br>- Abnormal vocalization<br>- Diurnal activity of nocturnal species	Suspected or Probable
"Possible"	The history of the rabies sings of the animal was unclear.<br>And<br>(The biting animal was killed/euthanized within 14 days post-bite<br>Or<br>The biting was stray animal with unknown post-bite details.)	
"Doubtful"	There was a history of an animal bite, and the animal that bit was likely still alive more than 14 days after the exposure. There are no other apparent animal bites	Not a case
"Unknown"	No bite histories or bite history is unclear	

*World Health Organization. WHO expert consultation on rabies: third report. Available from: https://apps.who.int/iris/handle/10665/

Supplementary Table S2.

Questioner of Knowledge, attitudes, and practices of patient

1 Does the patient can answer?	□No (go to 9) □Yes
2 Please describe rabies	
☐ Rabies as a disease ☐ Rabies described as change of behaviour of a dog/animal<br>☐ Unknown/wrong answer ☐ Declined to answer	
3 Please describe mode of transmission / how rabies can be caught?	
☐ Through bite or scratches ☐ Through scratches<br>☐ Unknown/wrong answer ☐ Declined to answer	
4 How severe is the disease called rabies?	
☐ Fatal nature of the disease known ☐ Fatal nature of the disease unknown ☐ Declined to answer	
5 What animals can be infected with rabies? Check all that apply (Patient answer)	
☐ Unknown ☐ Declined to answer ☐ Dog ☐ Cat ☐ Monkey<br>☐ Cattle ☐ Sheep ☐ Rodents ☐ Fox ☐ Bats<br>☐ Others (specify________________________)	
6 What would you do if you are bitten by a dog that you do not know/own? Check all that apply. (Patient answer)	
☐ None ☐ Wash wound ☐ Consult with a traditional healer (Tandoc)<br>☐ Apply home medics (Gallic / stone / papaya) ☐ Confine animal for observation<br>☐ Go to ABTC ☐ Submit animal for disease testing ☐ Kill animal<br>☐ Other (________________________) ☐ Declined to answer	
7 Do you know about ABTC? Do you know where is the nearest ABTC from your living place? (Patient answer)	
☐ Known collectedly ☐ Know wrongly ☐ Know ABTC but unknow the place of ABTC<br>☐ Do not know about ABTC ☐ Declined to answer	
8 Do you know the cost? (Patient answer)	
- Total cost in ABTC ……………….PHP / - Cost for vaccine …………… .PHP / Unknown<br>- Cost for RIG ……………….PHP / Unknown - Cost for transport …………….PHP/ Unknown<br>- Cost for other treatment (tetanus and antibiotics) ……………..PHP / Unknown	
Below the questions 9 ~14, if the patient can not answer, respondent (family) answer	
9 How long does it take time to go to the nearest ABTC?	……….min / □Unknown
10 Reasons for not seeking medical treatment. (multiple choice)	
☐ Patient did not know needed to go ☐ Cost ☐ It is not severe wound<br>☐ The dog was vaccinated ☐ I observed for 10days and the dog was alive<br>☐ Too far ABTC ☐ Patient had rabies vaccine ☐ Believe tandok<br>☐ Patient believe remedics (self-treatment) ☐ I did not know the location of ABTC<br>☐ Busy ☐ None ☐ Other reasons (___________________________)	

Laboratory methods. Standard Operation Procedure (SOP)

SOP. Collection saliva or swab samples from rabies suspected patients

In this SOP, we elucidate a protocol for the secure acquisition of saliva or swab samples from individuals diagnosed with rabies, followed by their prompt inactivation. It is imperative to adhere to safety protocols throughout the entire procedure. Prior to sample collection, informed consent must be secured from the patient or an appropriate family representative. Should the patient decline, the process must be terminated forthwith. It is recommended to utilize alcohol-based hand sanitizers consistently throughout the procedure. Furthermore, all staff involved should have received rabies vaccinations and must possess verified antibody titers (RFFIT) before undertaking this procedure.

To detail the procedure for collecting saliva and swab samples

Procedure for Saliva Collection:

Equipment Materials:

- PBS
- Swab (tongue clean mini mini. Men-tip®)
- 2.0ml Microcentrifuge tubes
- Pipette tips
- Biohazard Bag
- Screw-capped sample collection cup (for saliva)
- 1.5ml Microcentrifuge tube

Supplementary Figure 2. Swab (tongue clean mini mini. Men-tip®)

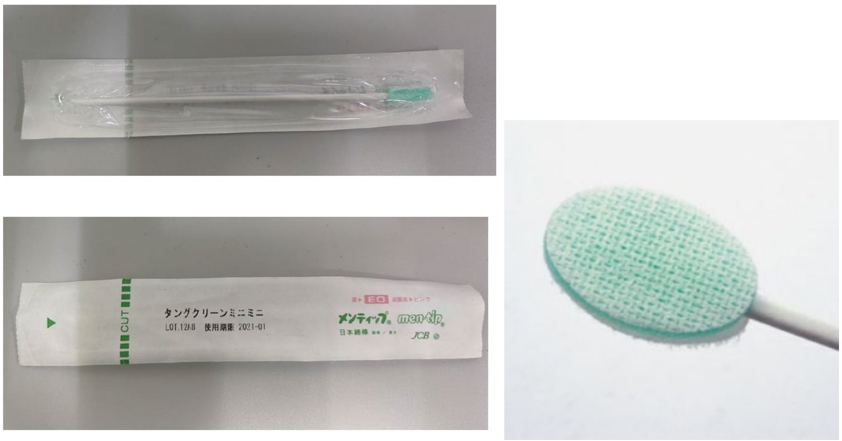

Procedure for Saliva Collection (Bedside procedure):

i. Begin by donning the appropriate Personal Protective Equipment (PPE). Once equipped, the research nurse should obtain the patient's consent and provide them with clear instructions regarding the collection process.

ii. Ask the patient to spit into the saliva collection cup. Ensure that the patient continues to spit until approximately 2ml of saliva has been collected.

iii. Carefully transfer 500µl of the collected saliva into a 2.0ml microcentrifuge tube. To this, add an equal volume (500µl) of PBS. Use a pipette to mix the solution by pipetting in and out.

iv. To ensure consistency and accuracy, repeat the saliva collection process two more times, ensuring there's an interval of 1 hour between each collection.

Procedure for saliva swab collection (Bedside procedure):

i. If the patient is unable to spit or produce saliva, take saliva swab sample using saliva swab (tongue clean mini mini. Men-tip®). Place the saliva swab in the patient's mouth, preferably under the tongue, and leave it there for around 10 seconds. Once saliva collects on the swab, remove it. If placing the swab proves challenging, perform scraping the oral mucosa, tongue, or sublingual area to collect the sample.

ii. Once the swab is taken, place the swab in a 2.0ml microcentrifuge tube containing 500µl of PBS. Ensure that the swab is mixed in the buffer for at least 10 seconds.

iii. Twist the swab against the side of the tube to remove any excess fluid. After ensuring maximum fluid transfer, discard the swab.

iv. Label the tube appropriately to ensure proper identification. Store the sample immediately at −30°C after collection.

v. For consistency and thorough sampling, repeat the swab collection process two more times, ensuring there's an interval of 1 hour between each collection.

SOP. RNA extraction from saliva or swab samples

Reagents and kits:

- Roche High Pure Viral Isolation Kit
- Absolute Ethanol (reagent grade)
- Prepare in advance: Lysis Binding buffer supplemented with Carrier RNA
  (1ml of Binding buffer + 10µl poly A carrier RNA)

Equipment:

Centrifuge

Vortex

Specimen:

Saliva buffer sample: Saliva or swab samples are taken bedside and then mixed with a buffer solution.

RNA extraction procedure:

i. Begin by mixing 200µl of saliva buffer samples with 400µl of Binding Buffer that contains Carrier RNA to deactivate the sample. (Biosafety cabinet).

ii. Allow the mixture to incubate at room temperature, ranging between 15°C and 25°C, for a duration of 10 minutes.

iii. Carefully transfer the mixture into the High Pure Filter column, ensuring not to exceed 700µl per application. Once done, insert the column into a collection tube.

iv. Centrifuge the tube setup for 15 seconds at a speed of 8,000 × g. After centrifugation, discard the flow-through.

v. Introduce 500µl of Inhibitor Removal Buffer into the column.

vi. Proceed to centrifuge for 1 minute at 8,000 × g. Once again, discard the flow-through post-centrifugation.

vii. Add 450µl of Wash Buffer to the column and centrifuge for 1 minute at 8,000 × g. Discard the flow-through after this step.

viii. Repeat the addition of 450µl of Wash Buffer and centrifuge for 1 minute at 8,000 × g. To ensure the removal of any residual buffer, centrifuge once more for 10 seconds at maximum speed.

ix. Transfer the filter column to a freshly labeled microcentrifuge tube.

x. Add 50µl of elution buffer to the column.

xi. Centrifuge the setup for 1 minute at 8000 × g to elute the RNA.

xii. Store the eluted RNA at −80°C for future use.

1.1 SOP N sequencing method

Genome sequencing aids in the understanding of the molecular epidemiology and surveillance of the rabies virus in the country.

- Reagents and Equipment

Takara PrimeScript One Step RT-PCR Kit

Absolute Ethanol

Microtube centrifuge

Thermal cycler

Electrophoresis chamber

UV light Irradiation apparatus

- Primers

primer name	seq	for	Region	Location
p1	ACAGACAGCGTCAATTGCAAAGC	PCR/seq	N	28-50
304	TTGACGAAGATCTTGCTCAT	PCR/seq	N	1514-1533

- Procedure for one step-PCR

i. Reagent Preparation Area:

Begin by moving to the designated area for reagent preparation.

ii. Tube Labeling:

Label tubes for RT-PCR reactions. Arrange the reaction tubes starting with the samples. Intersperse a negative control tube every three samples to monitor for potential cross-contamination. Allocate the second-to-the-last tube for the positive control.

iii. RNA Sample Thawing:

Thaw the RNA samples. Once thawed, briefly spin the tubes in a microcentrifuge to collect the RNA at the bottom. Immediately place the tubes on ice after spinning.

iv. RT-PCR Mastermix Preparation:

Prepare the RT-PCR mastermix using the Takara PrimeScript One Step RT-PCR Kit. Ensure all work is performed on ice.

Each reaction should use a total volume of 25µl. Include an additional preparation to account for pipetting errors.

Calculate the total amount of each reagent required for the RT-PCR mastermix based on the number of reactions plus the extra preparation.

Aliquot 22.5µl of the prepared mastermix into each of the previously labeled tubes.

Add 2.5µl of the extracted RNA sample to its corresponding tube.

Carefully move to the designated area for template addition and nucleic acid extraction.

Add 2.5µl of RNase-free water to the negative control tubes.

Add 0.5µl of control RNA to the positive control tubes. To achieve the required total reaction volume of 25µl, add an additional 2.0µl of RNase-free water to these tubes.

Proceed to the PCR area with the prepared tubes.

Load the reaction tubes into the thermocycler.

Run the following protocol (specific cycling conditions should be inputted based on the kit's instructions and your experimental design):

**Reagent**	**1* reaction volume (µL)**
dH2O	7
2x one-step buffure	12.5
Forward Primer P1 (10µL)	12.5
Reverse Primer 304 (1 µL)	12.5
PrimeScript 1step enzyme mix (5µL)	0.5
RNA template	2.5

**Temperature (C°)**	**Time**	**No. of cycles**
50	30 min	1
94	2 min	1
94	15 sec	40
50	30 sec	1
68	2min	1
68	5min	1
4	-	

v. Gel Electrophoresis

To confirm the presence of the target gene (expected size: 1506bp) using agarose gel electrophoresis.

Electrophoresis Procedure for Target Gene Confirmation

Prepare a 1.5% Agarose Gel: Dissolve agarose in buffer, pour into a casting tray, and let solidify.

Load PCR Products: Place the gel in the electrophoresis chamber, load PCR products and a DNA ladder into the wells.

Stain the Gel: Use Ethidium Bromide (EtBr) or SYBR Safe for staining.

Run Electrophoresis: Conduct electrophoresis at an appropriate voltage until the dye front is adequately migrated.

UV Visualization: Visualize the gel under UV light to confirm the presence of a 1506bp band.

vi. PCR:

Ensure that only PCR products displaying a single clear band corresponding to the expected base pair size are selected for sequencing. These bands should be distinct with no nonspecific bands present.

vii. Handling Nonspecific Bands:

If nonspecific bands are observed, refer to a separate Standard Operating Procedure (SOP) for QIAquick Gel Extraction using a Microcentrifuge to purify the desired product.

viii. Sample Preparation for Sequencing:

Follow the designated SOP for sample preparation specific to sequencing requirements.

Submission to sanger sequence: Once prepared according to the SOP, keep the PCR products and primers safely

stored and send them for genome sequencing. Genome sequencing was performed by Kinovett company, Philippines (https://www.kinovett.com/).

ix. Additional Primers for Sequencing:

We used a cocktail of JW6 DPL (Duvenhage virus, rabies virus strain Pasteur, and Lagos bat virus) (CAA TTC GCA CAC ATT TTG TG), JW6 M (Mokola virus)(CAG TTA GCG CAC ATC TTA TG) and JW6 E (European bat lyssavirus-1 and -2) (CAG TTG GCA CAC ATC TTG TG) primers for sequencing. Sequencing was performed by Ma

x. Phylogenetic Analysis of N Gene Sequences

- Use GeneStudio version 2.2.0.0 (available at GeneStudio) to trim and edit the original sequences obtained from the RT-PCR assay.
- Align the trimmed sequences to the reference sequence (GenBank accession no. NC_001542.1).
- Perform multiple-sequence alignment of the N gene sequences using Molecular Evolutionary Genetics Analysis version X (MEGA X), available at MEGA Software.
- Construct the phylogenetic tree using the maximum likelihood method in MEGA X.
- Calculate the bootstrap probability from 500 replicates for tree reliability assessment.
- Select the Tamura 3-parameter with gamma distribution model as the substitution model. This choice should be based on the Akaike information criterion with a correction value in the model selection feature of MEGA X.
- Set the cutoff value for the condensed tree at 70%.
- Include additional reference sequences with the genetic clades assigned by RABV-GLUE (http://rabv-glue.cvr.gla.ac.uk/#/project/alignment accessed on 1 February 2024) obtained from GenBank (NCBI GenBank).
- Compare the sequences to those of other rabies lyssaviruses in the Philippines and Asia.
- These reference sequences used were obtained from the GenBank database (https://www.ncbi.nlm.nih.gov/search/).

Supplementary Table S3. Reference sequences used in the study

**Country**	**Isolate ID**	**Clade**	**Host**	**Year**	**Gene**	**Accession No.**
China	RV_J	Asian SEA1a	*Homo sapiens*	1986	N	GU345747
China	98011CHI	Asian SEA1b	*Canis familiaris*	1998	N	KX148265
China	GX4	Asian SEA2a	*Canis familiaris*	1994	N	GU358653
China	JX08_45	Asian SEA2b	*Melogale moschata*	2008	N	GU647092
Thailand	8764THA	Asian SEA3	*Homo sapiens*	1983	N	EU293111
Philippines	94275PHI	Asian SEA4	*Canis familiaris*	1994	N	KX148263
Taiwan	R2012_88	Asian SEA5	*Melogale moschata*	2012	N	KF620488

Reference:

Heaton PR, Johnstone P, McElhinney LM, Cowley R, O'Sullivan E, Whitby JE. Heminested PCR assay for detection of six genotypes of rabies and rabies-related viruses. J Clin Microbiol. 1997;35: 2762–2766. doi:10.1128/jcm.35.11.2762-2766.1997

Supplementary Figure S3.
Monthly rabies admissions and study enrollments at San Lazaro Hospital during the study period

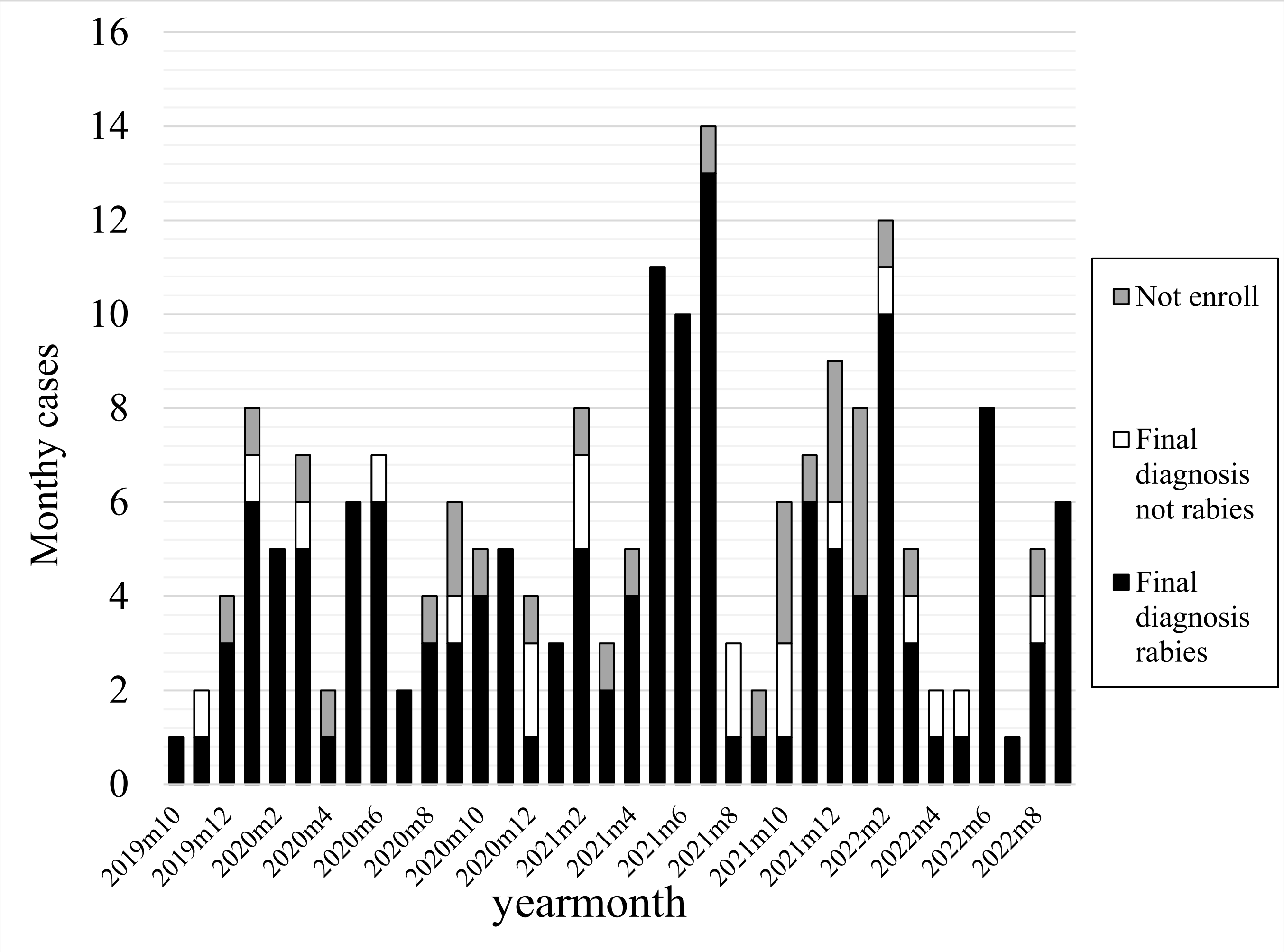

A final (discharge) diagnosis of rabies is made if the patient exhibits symptoms of rabies, including hydrophobia and aerophobia, and dies within several days of admission. 28 patients with final diagnosis of rabies were not included in the study due to either their death before the research staff was not able to approach them or the staff's inability to enroll them.

Supplementary Table S4
Comparison between two previous retrospective studies (1987-2006, 2006-2011) and the prospective study (October 2019 - September 2022)

		1987–2006 (n=1839)	2006–2011 (n=463)	**Oct 2019–Sep 2022 (n=151)**
Yearly cases, mean (range)		92 (57-119)	57.5 (35-72)	**60.0***
Sex %	Male	68.7%	70.3%	**75.5%**
	Female	31.2%	29.7%	**24.5%**
Age group %	<5	33.3%	3.8%	**2.0%**
	5-19		20.9%	**21.2%**
	>20	66.6%	75.3%	**76.8%**
Living area %	NCR		28.0%	**18.5%**
	Region III		34.4%	**47.7%**
	Region IVA		29.4%	**35.1%**
Causal animals %	Dog	97.1%	96.3%	**97.0%**
	Cat	2.9%	3.7%	**3.0%**
Causal animal: Pet or stray animals %	Pet	35.5%	25.9%	**60.2%**
	Stray	64.5%	17.8%	**33.1%**
	Unknown		59.1%	**6.7%**
Characteristics of the exposure %	Bite	98.6%	95.0%	**91.0%**
	Scratch		2.3%	**3.8%**
	Lick		1.6%	**0.8%**
Type of the exposure %	Sigle		74.7%	**69.2%**
	Multiple		3.1%	**30.8%**
	Unknown		22.2%	**0.0%**
Incubation period %	<30	16.0%	22.7%	**15.0%**
	30-90	27.3%	42.1%	**53.4%**
	91-365	43.0%	18.5%	**21.1%**
	>365	13.7%	9.4%	**7.5%**
	Unknown		7.6%	**0.0%**
Rabies vaccines and RIG %	None	98.3%	90.4%	**93.2%**
	At least 1 vaccine	1.7%	9.6%	**6.8%**
	Complete PEP		0.5%	**2.3%**

* The number have been adjusted to reflect an annual average over the three-year study period. In addition to the enrolled rabies patients, 28 patients who were admitted to SLH with a final diagnosis of rabies but could not be registered in the study are also included.

Supplementary Table S5

Clinical symptoms, hospital treatments, and intervals from illness onset to hospital outcomes among rabies patients with a final diagnosis of rabies (n=151)

		N (%)
Symptoms	Bite site symptoms (Pain or Numbness)	23 (15.2)
	fever	57 (37.8)
	Headache	19 (12.6)
	Malaise or fatigue	122 (80.8)
	Sore throat	32 (21.2)
	Chest pain	9 (6.0)
	Vomiting / Nausea	108 (71.5)
	Loss of appetite	136 (90.1)
	Abdominal pain	12 (8.0)
	Difficult breathing	139 (92.1)
	Behavioural change	138 (91.4)
	Restlessness	144 (95.4)
	Agitation or aggression	140 (92.7)
	Insomnia	80 (53.0)
	Anxiety	115 (76.2)
	Stiff neck	1 (0.7)
	Confusion / coma	8 (5.3)
	Paralysis/paresis	0 (0.0)
	Seizure	0 (0.0)
	Aerophobia	145 (96.0)
	Hydrophobia	151 (100.0)
	Hypersalivation	55 (36.4)
	Photophobia	15 (9.9)
Signs and status during the admission	Fever (38°C) or greater	42 (27.8)
	Shock	21 (13.9)
	GCS <15	36 (23.8)
	SpO2 <90%	32 (21.2)
Treatment	IV fluid	5 (3.3)
	O2	0 (0.0)
	Ventilator	0 (0.0)
	Diazepam	119 (78.8)
	Diphenhydramine	116 (76.8)
Type of rabies	Furious rabies	151 (100.0)
	Paralytic (or dumb) rabies	0 (0.0)

Time interval from illness onset to hospital admission (Median 2 days, Range 0–6 days)	0–1 day<br>2–3 days<br>≥ 4days	51 (33.8)<br>77 (51.0)<br>23 (15.2)
Time interval from illness onset to death (Median 3 days, Range 0–8 days)	0–1 day<br>2–3 days<br>≥ 4days	22 (14.6)<br>86 (57.0)<br>43 (28.5)
Time interval from hospital admission to death (Median 11.7 hours, Range 0.1–68.3 hours)	< 24 hours<br>24–48 hours<br>49–72 hours	120 (79.5)<br>27 (17.9)<br>4 (2.7)

Supplementary Figure S4.
Distribution of incubation periods among rabies patients with a history of animal exposure (n=133).

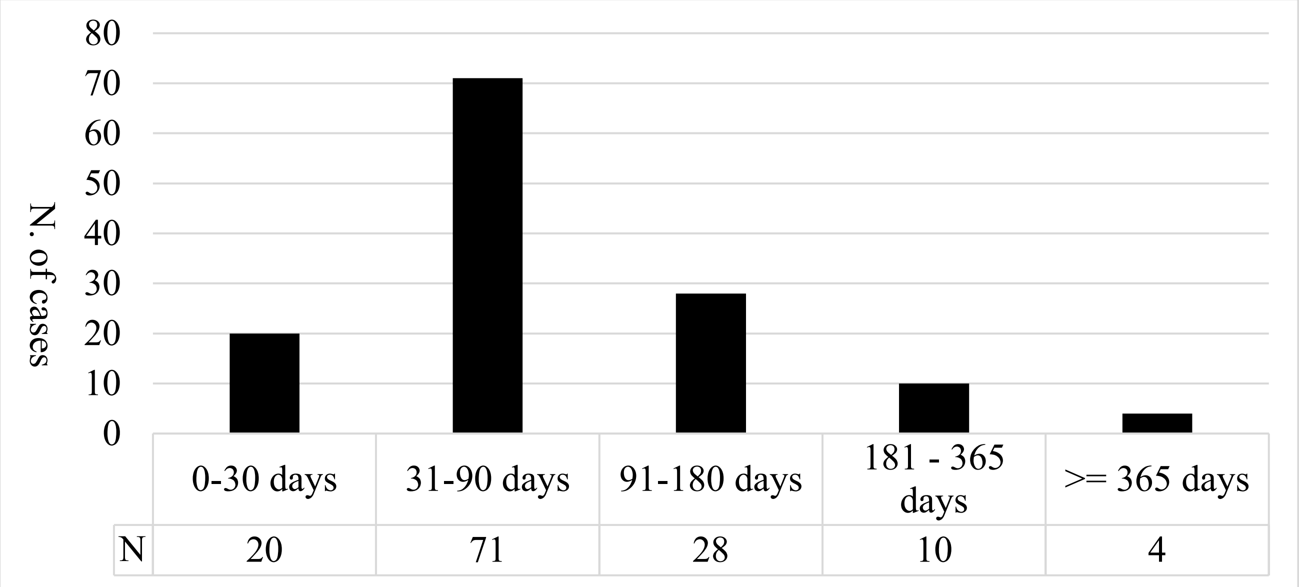

This analysis includes only patients with a probable or possible history of animal exposure that led to rabies. The incubation period is calculated as the number of days between the exposure to the animal and the onset of symptoms.

Supplementary Table S6.

Relationship between incubation period and bitten body parts

N (%)	Total	0-30 days	31-90 days	91-180 days	181-365 days	>=1 year
Lower limb	48	2 (4.2)	26 (54.2)	13 (27.1)	4 (8.3)	3 (6.3)
Hand	56	7 (12.5)	34 (60.7)	11 (19.6)	3 (5.4)	1 (1.8)
Upper limb	11	1 (9.1)	5 (45.5)	3 (27.3)	2 (18.2)	0 (0)
Trunk	1	0 (0)	0 (0)	0 (0)	1 (100)	0 (0)
Head or neck	17	10 (58.8)	6 (35.3)	1 (5.9)	0 (0)	0 (0)

The closest body part to the head was selected when the patient had multiple bites.

Supplementary Table S7.
Sensitivity and specificity of LN34-qRT-PCR using single, double, or triple saliva/swab samples.

		Final diagnosis rabies	Final diagnosis not rabies	Sensitivity (95%CI)	Specificity (95%CI)
Single (n=171)	Posi	70	0	46.4%	100%
	Neg	81	20	(38.2–54.6)	(83.2–100)
Double (n=164)	Posi	86	0	59.7%	100%
	Neg	58	20	(51.2–67.8)	(83.2–100)
Triple (n=161)	Posi	95	0	67.4%	100
	Neg	46	20	(59.0–75.0)	(83.2–100)

Posi, Positive. Neg, Negative

We collected saliva samples from patients who was able to produce saliva, while we used saliva swab from those who were not able to produce saliva samples. The collection methods and testing procedures are detailed in the supplementary materials. A minimum interval of one hour was maintained between each sample collection. For the single sample analysis, only the first of consecutive samples was analyzed. For the double, both the first and second samples were analyzed together; a positive result in either was considered positive, and negative if both were negative. If the sample is positive on the first sample, it is considered positive for double even if there are no second sample. The triple required three sample collections, with any one positive result deemed positive. If the sample is positive on either the first sample or second sample, it is considered positive for triple even if there are no second or third sample. The most common reason for not being able to collect the second and third samples was patient death following the first sample collection.

Supplementary Table S8.
Sensitivity and specificity by sample type (Saliva or Swab) of LN34 RT-qPCR

Sample type	Total N. (Positive/Negative)	Total N. 1st sample	Total N. 2nd sample	Total N. 3rd sample	Sensitivity % (95%CI)	Specificity % (95%CI)
Saliva	244 (87/157)	96	83	65	41.6 (34.9-48.6)	100 (90-100)
Swab	225 (107/118)	75	74	76	53.5 (46.3-60.6)	100 (86.3-100)
Total*	469 (194/275)	171	157	141	47.4 (42.5-52.4)	100 (94-100)

We obtained a total of 449 samples from 171 patients (151 final diagnosed with rabies, 20 diagnosed with other diseases). Swabs were used for patients unable to produce saliva (due to severe illness or dehydration). Calculate sensitivity and specificity based on the final diagnosis as the reference diagnostic method. It may not be appropriate as there is a high likelihood of severe cases (end-stage) being included in the swab sample group, making simple comparisons between these sample types difficult. *This result indicates the sensitivity of one sample. The sensitivity mentioned in the main text and Supplementary 10 represents the sensitivity based on multiple samples (positive if any sample is positive).

Supplementary Table S9.
Rapid Fluorescent Focus Inhibition Test (RFFIT) results by the frequencies of rabies vaccinations

	Under detectable level <0.05 IU/ml	0.05–0.499 IU/ml	≥ 0.5 IU/ml	No samples
No previous vaccinations	101	11	1	24
Had a previous PrEP or PEP* before the bite exposure to leading to rabies	5	0	0	0
One dose of vaccination as PEP	1	0	0	0
Two doses of vaccinations as PEP	1	0	0	1
Three doses of vaccinations as PEP	0	0	6	0

* No one received PEP among individuals with a history of previous PrEP or PEP before the animal bite exposure that led to rabies.
PrEP, pre-exposure prophylaxis. PEP, Post exposure prophylaxis.

Supplementary Table S10.
Additional information of Table 2. Outcomes of the biting animals, rabies tests, wrong home treatments, and treatments by traditional healer

		n (%)
Animal status 10 days after the bite incidence	Died naturally	44 (33.1)
	Killed or Euthanasia	48 (36.1)
	Unknown	41 (30.8)
Request rabies test for the dead animal (n=92)	No test	89 (96.7)
	Unknown	3 (3.3)
Wrong home medication (n=77)	Garlic	23 (17.3)
	Sucking on a wound	50 (37.6)
Treatments by traditional healer (n=44)	Cut or suck bite sites	27 (61.4)
	Traditional herb medicine	2 (4.6)
	Stone	6 (13.6)

Supplementary Table S11
Reasons patients did not receive rabies vaccination and immunoglobulin at medical facilities.

Study ID	Frequency of Vac and RIG	Reason why rabies vaccination and RIG was not given at the medical facilities
6	0 Vac + 0 RIG	The patient visited ABTC, but the anti-rabies vaccination was unavailable; therefore, they received only an anti-tetanus vaccination. Subsequently, they were advised to visit a private hospital for the rabies vaccine, but the family lacked sufficient funds to afford it
58	0 Vac + 0 RIG	The patient received only tetanus vaccine because the patient did not have sufficient funds to purchase the anti-rabies vaccine.
77	0 Vac + 0 RIG	The anti-rabies vaccine was not available at the nearest ABTC where the patient visited. The family was instructed to visit another hospital, but the family lacked the necessary money, and the community lockdown restrictions further prohibited them from leaving the area.
81	0 Vac + 0 RIG	The anti-rabies vaccine and RIG were not available at the nearest ABTC, and the patient was advised to purchase it externally for approximately 4,000 PHP. Lacking sufficient funds, the patient opted to visit a traditional healer for a cost of 200 PHP instead.
90	0 Vac + 0 RIG	The anti-rabies vaccine was out of stock when the patient visited, and only an anti-tetanus vaccination was administered. The patient was advised to return the following day for the anti-rabies vaccine but did not come back due to work obligations.
93	0 Vac + 0 RIG	The patient was administered only a tetanus toxoid vaccine, and the family was unaware of the reason for not receiving any rabies vaccinations
111	0 Vac + 0 RIG	Due to the unavailability of the anti-rabies vaccination at the initial hospital, the patient was directed to a different medical facility. However, the higher cost of the vaccine there led the family to decide against seeking treatment at this alternative location.
117	0 Vac + 0 RIG	The patient received only a tetanus toxoid vaccine, as the anti-rabies vaccine was not available at the health cener. The staff requested the patient's contact number, intending to call when the public health center had the anti-rabies vaccination in stock.
125	0 Vac + 0 RIG	The patient went to the hospital for an anti-rabies vaccination, but was informed that at least four animal bite patients were required before a vaccine vial could be opened. As a result, the patient eventually failed to receive the vaccine.
Reason why RIG (Rabies immunoglobulin) was not given at the medical facilities		

20	3Vac + 0 RIG	The patient visited the ABTC and received three doses of anti-rabies vaccine, anti-tetanus, and antibiotics. However, RIG was not available at the health center, and they were referred to a private hospital for RIG. Unfortunately, they did not have sufficient funds to afford RIG at the private hospital.
46	3Vac + 0 RIG	The patient visited a hospital and received an anti-rabies vaccination. Since RIG was not available, they were referred to another hospital. The doctor at the other hospital advised that RIG should be administered on the 30th day of the anti-rabies vaccination schedule.
65	1Vac + 0 RIG	The patient was not able to receive RIG due to the cost of RIG (4,000php). Also due to the lockdown, the patient was not able to visit medical facility for following rabies vaccines.

RIG, rabies immunoglobulin. ERIG, Equine RIG. Vac, vaccination

Supplementary Table S12

Covid19 pandemic related factors and reasons patients were unable to visit health facilities.

Study ID	Factors and Reasons
28	Due to the lockdown, the patient was restricted from going outside, resulting in their inability to receive the third dose of the anti-rabies vaccination.
33	There was no available transportation to the ABTC during the lockdown, preventing the patient from reaching the centre for treatment.
36	During the lockdown, health centres were closed due to COVID-19 precautions. Additionally, patients often did not seek treatment, considering their minor bite injuries as non-critical.
39	Due to the lockdown in their community, the patient sought treatment from a nearest traditional healer rather than health facilities.
47	The patient was hesitant to go outside due to fears due to COVID-19 pandemic, and they also thought that treatment was not unnecessary because the bite wound was not severe.
53	Due to the community lockdown, residents were not permitted to go outside. The fear of contracting COVID-19 was heightened as there was a confirmed positive case within their community.
65	Owing to the lockdown, the patient was unable to leave for the third dose and thus received only two doses of the anti-rabies vaccination.
74	All senior citizens were not allowed to go outside due to COVID-19.
77	Because of lockdown in their community, they were not allowed to go outside

Supplementary Table S13. STROBE Statement—checklist of items that should be included in reports of observational studies.

	Item No.	Recommendation	Page No.	Relevant text from manuscript
Title and abstract	1	(*a*) Indicate the study's design with a commonly used term in the title or the abstract	P1(Title), P4 (abstract)	
		(*b*) Provide in the abstract an informative and balanced summary of what was done and what was found	P4–5 (abstract)	
Introduction				
Background/rationale	2	Explain the scientific background and rationale for the investigation being reported	P8-9 Introduction	
Objectives	3	State specific objectives, including any prespecified hypotheses	P 9	"To address…"
Methods				
Study design	4	Present key elements of study design early in the paper	P11	"We conducted a…"
Setting	5	Describe the setting, locations, and relevant dates, including periods of recruitment, exposure, follow-up, and data collection	P10–11	
Participants	6	(*a*) *Cohort study*—Give the eligibility criteria, and the sources and methods of selection of participants. Describe methods of follow-up *Case-control study*—Give the eligibility criteria, and the sources and methods of case ascertainment and control selection. Give the rationale for the choice of cases and controls *Cross-sectional study*—Give the eligibility criteria, and the sources and methods of selection of participants	P11−12	
		(*b*) *Cohort study*—For matched studies, give matching criteria and number of exposed and unexposed	NA	

		Case-control study—For matched studies, give matching criteria and the number of controls per case	
Variables	7	Clearly define all outcomes, exposures, predictors, potential confounders, and effect modifiers. Give diagnostic criteria, if applicable	NA
Data sources/ measurement	8	For each variable of interest, give sources of data and details of methods of assessment (measurement). Describe comparability of assessment methods if there is more than one group	NA
Bias	9	Describe any efforts to address potential sources of bias	NA
Study size	10	Explain how the study size was arrived at	NA

Quantitative variables	11	Explain how quantitative variables were handled in the analyses. If applicable, describe which groupings were chosen and why	NA
Statistical methods	12	(*a*) Describe all statistical methods, including those used to control for confounding	P12–13
		(*b*) Describe any methods used to examine subgroups and interactions	NA
		(*c*) Explain how missing data were addressed	NA
		(*d*) *Cohort study*—If applicable, explain how loss to follow-up was addressed<br>*Case-control study*—If applicable, explain how matching of cases and controls was addressed<br>*Cross-sectional study*—If applicable, describe analytical methods taking account of sampling strategy	NA
		(*e*) Describe any sensitivity analyses	

Results

Participants	13	(a) Report numbers of individuals at each stage of study—eg numbers potentially eligible, examined for eligibility, confirmed eligible, included in the study, completing follow-up, and analysed	Figure 1	
		(b) Give reasons for non-participation at each stage	P 14	"During the study.."
		(c) Consider use of a flow diagram	Figure 1	
Descriptive data	14	(a) Give characteristics of study participants (eg demographic, clinical, social) and information on exposures and potential confounders	Table 1	
		(b) Indicate number of participants with missing data for each variable of interest	NA	
		(c) *Cohort study*—Summarise follow-up time (eg, average and total amount)	NA	
Outcome data	15	*Cohort study*—Report numbers of outcome events or summary measures over time	NA	
		Case-control study—Report numbers in each exposure category, or summary measures of exposure	NA	
		Cross-sectional study—Report numbers of outcome events or summary measures	NA	
Main results	16	(*a*) Give unadjusted estimates and, if applicable, confounder-adjusted estimates and their precision (eg, 95% confidence interval). Make clear which confounders were adjusted for and why they were included	NA	
		(*b*) Report category boundaries when continuous variables were categorized	Yes	
		(*c*) If relevant, consider translating estimates of relative risk into absolute risk for a meaningful time period	NA	

NA, Not applicable
